# Mechanisms behind altered pulsatile intracranial pressure in idiopathic normal pressure hydrocephalus: role of vascular pulsatility and systemic hemodynamic variables

**DOI:** 10.1007/s00701-020-04423-5

**Published:** 2020-06-12

**Authors:** Karen Brastad Evensen, Per Kristian Eide

**Affiliations:** 1 Department of Neurosurgery, Oslo University Hospital – Rikshospitalet, P.O.Box 4950, Nydalen, 0424 Oslo, Norway; 2 Department of Informatics, Faculty of Mathematics and Natural Sciences, University of Oslo, Oslo, Norway; 3 Institute of Clinical Medicine, Faculty of Medicine, University of Oslo, Oslo, Norway

**Keywords:** ICP wave amplitudes, Central aortic waveforms, Idiopathic normal pressure hydrocephalus, Autoregulation

## Abstract

**Background:**

The dementia subtype idiopathic normal pressure hydrocephalus (iNPH) has unknown etiology, but one characteristic is elevated intracranial pressure (ICP) wave amplitudes in those individuals who respond with clinical improvement following cerebrospinal fluid (CSF) diversion. To explore the mechanisms behind altered ICP wave amplitudes, we correlated central aortic blood pressure (BP) and ICP waveform amplitudes (intracranial aortic amplitude correlation) and examined how this correlation relates to ICP wave amplitude levels and systemic hemodynamic parameters.

**Methods:**

The study included 29 patients with probable iNPH who underwent continuous multi-hour measurement of ICP, radial artery BP, and systemic hemodynamic parameters. The radial artery BP waveforms were used to estimate central aortic BP waveforms, and the intracranial aortic amplitude correlation was determined over consecutive 4-min periods.

**Results:**

The average intracranial aortic amplitude correlation was 0.28 ± 0.16 at the group level. In the majority of iNPH patients, the intracranial aortic amplitude correlation was low, while in about 1/5 patients, the correlation was rather high (average Pearson correlation coefficient > 0.4). The degree of correlation was hardly influenced by systemic hemodynamic parameters.

**Conclusions:**

In about 1/5 iNPH patients of this study, the intracranial aortic amplitude correlation (IAAC_AORTIC_) was rather high (average Pearson correlation coefficient > 0.4), suggesting that cerebrovascular factors to some extent may affect the ICP wave amplitudes in a subset of patients. However, in 14/19 (74%) iNPH patients with elevated ICP wave amplitudes, the intracranial aortic amplitude correlation was low, indicating that the ICP pulse amplitude in most iNPH patients is independent of central vascular excitation, ergo it is modulated by local cerebrospinal physiology. In support of this assumption, the intracranial aortic amplitude correlation was not related to most systemic hemodynamic variables. An exception was found for a subgroup of the patients with high systemic vascular resistance, where there was a correlation.

## Introduction

Idiopathic normal pressure hydrocephalus (iNPH) is a subtype of dementia incorporating gait ataxia, urinary incontinence, and cerebrospinal fluid (CSF) circulation failure, but with an unknown cause. We have previously reported that the iNPH patients responding clinically to CSF diversion surgery (shunt surgery) typically present with elevated ICP wave amplitudes measured invasively [7, 8]. While elevated ICP wave amplitudes characterize iNPH shunt responders, the underlying mechanisms remains unclear. For example, are the ICP wave amplitudes primarily related to vascular factors such as the blood pressure (BP) wave amplitudes or are the ICP wave amplitudes more influenced by extravascular factors?

In particular, the association between pulsatile arterial BP and ICP may be one factor behind the delayed paravascular clearance of CSF tracer observed in vivo in individuals with iNPH [6, 34]. In 2012, a brain-wide paravascular route for transport of fluids and solutes, denoted the glymphatic (glia-lymphatic) system, was described [15]. Convective forces created by the pressure gradients from the arterial pulsatile BP were hypothesized to represent the primary driving force behind the antegrade transport of fluid and solutes along the blood vessels [15, 16, 24]. Moreover, reduced arterial pulsations, such as seen in arterial hypertension, are associated with hampered paravascular solute transport [24]. With regard to iNPH, we have proposed that restricted arterial BP pulsatility related to abnormal pulsatile ICP may hamper the paravascular waste removal [6].

**Fig. 1:**
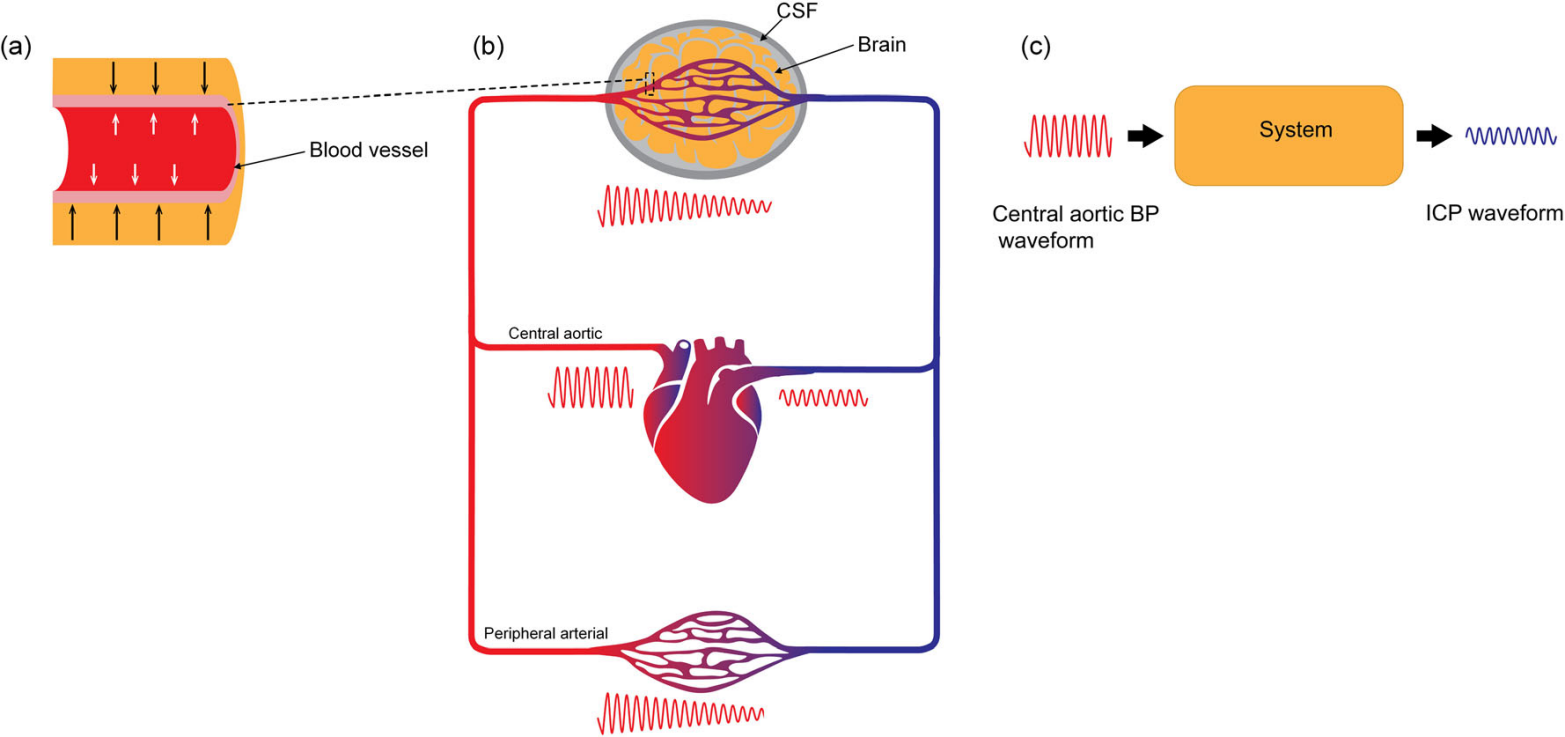
The transmission from central aortic BP waveforms to ICP waveforms. **a** The pulsatile arterial BP causes pressure forces in the radial direction (white arrows) and towards the arterial blood vessel wall. The degree of movement of the arterial blood vessel wall partly depends on the properties of arterial wall (e.g., stiff blood pressure walls as seen in arterial hypertension, auto-regulatory capacity) and the blood flow (here collectively denoted *vascular factor*), and partly on the counter pressure (black arrows) determined by factors in the compartment outside the blood vessel (here denoted *extra-cerebrovascular factor*). **b** The cardiac contractions create arterial BP waveforms that propagate via the systemic circulation and cerebral circulation. Typically, the arterial BP waveform diminishes from the arterial (red) to the venous side (blue). While it is not feasible to continuously monitor the pulsatile arterial BP within the cranial compartment, the central aortic BP waveform is more closely related to the intracranial arterial BP waveform than peripheral arterial BP, e.g., the radial arterial BP waveform. **c** In this study, the “unknown” system consists of the *vascular factors* (intracranial blood vessels and cerebral blood flow) and *extra-cerebrovascular factors* (brain parenchyma and CSF). The central aortic BP waveform acts as a proxy for the intracranial arterial BP waveform and is our input signal, while the measured ICP waveform is the output signal. To study to which extent the vascular and extra-cerebrovascular factors constitute the system, we determined the Pearson correlation coefficient between amplitudes of central aortic and ICP waveforms for every 4 min period (referred to as Intracranial Aortic Amplitude Correlation, IAAC_AORTIC_). A high degree of correlation between input and output suggests a semi-direct transfer from input to output, i.e., ICP is mainly determined by vascular factors (arterial BP) and the extra-cerebrovascular factors of the system are negligible. A low correlation indicates that the extra-cerebrovascular factors of the system highly affect the measured ICP signal. Illustration: Ine Eriksen, University of Oslo

The present study addressed to which degree the pulsatile ICP that is created from the pulsatile arterial BP is affected by extra-cerebrovascular factors in iNPH patients. As a surrogate marker of arterial BP pulsatility within the cranial cavity, we utilize the central aortic BP waveforms, which are close to the scene both for hemodynamic events and the intracranial arterial pulsations. The concept described here is illustrated in Fig. 1. Using terms from system analysis, we consider the pulsatile central aortic BP our input signal and the pulsatile ICP our output signal. The unknown system consists of the vascular (blood vessel and flow) and extra-cerebrovascular factors (brain parenchyma and CSF) and will act as a physiological filter on the central aortic BP waves. The measured ICP waves are then the final result. This yields that the correlation between central aortic BP and ICP waveforms, here denoted *I*ntracranial *A*ortic *A*mplitude *C*orrelation (IAAC_AORTIC_), provides information about the impact of extra-cerebrovascular factors on the ICP waveform. A high degree of correlation would suggest a direct transfer of the central aortic BP waveform to the ICP waveform (i.e., extra-cerebrovascular compartment hardly affects the ICP waveform). If the abnormal ICP waveform in iNPH patients primarily is determined by vascular BP pulsatility, we would expect IAAC_AORTIC_ to be increased and to be affected by systemic vascular variables and various patient characteristics. We would, however, not expect that surrogate markers of the extra-cerebrovascular content to affect the correlation. Our hypothesis is, therefore, that the transfer of central aortic BP to ICP waveform primarily is affected by extra-cerebrovascular factors and that the elevated pulsatile ICP seen in iNPH patients are due to other pathophysiological factors than vascular factors per se.

## Materials and methods

### Patients

The study included patients with *probable* iNPH [33] who underwent work-out for CSF diversion surgery at the Department of Neurosurgery, Oslo University Hospital—Rikshospitalet, from October 2008 to January 2009. Multi-hour continuous ICP monitoring with the determination of mean ICP wave amplitude (MWA_ICP_) is part of the clinical routine and an important factor when deciding on whether to perform CSF diversion surgery. Individuals with mean ICP wave amplitudes (MWA_ICP_) above a selected threshold are offered shunt surgery, as previously described [7].

### Monitoring and analysis of continuous pressure and hemodynamic variables

The ICP was measured continuously using a solid ICP sensor (Codman MicroSensor™, Johnson & Johnson, Raynham, MA, USA) that had been placed 1–2 cm into the frontal brain parenchyma through a small burr hole and a minimal opening in the dura in local anesthesia. The radial artery BP was measured continuously and invasively from the right radial artery using a Truwave PX-600F Pressure Monitoring Set (Edwards Life sciences LLC, Irvine, CA) that was placed at the level of the heart. Both the ICP waveform and radial artery BP waveforms were sampled at 200 Hz, which is an adequate sampling rate [14] and digitized using an analogue-to-digital converter (Sensometrics® Pressure Logger; dPCom AS, Oslo, Norway) before they were stored as raw data files with an identical time reference. The continuous pressure signals were analyzed using Sensometrics® software (dPCom AS, Oslo, Norway).

Central aortic BP waveforms were estimated from the radial artery BP waveforms using the SphygmoCor system (SphygmoCor®; AtCor Medical, West Ryde, NSW, Australia). The SphygmoCor system has been validated to successfully estimate central aortic BP waveforms from several different variants of peripheral BP measurements. The most relevant validation studies are brachial artery BP measurements [17], invasive radial artery BP measurements [25], and radial artery tonometry measurements [3, 12]. In addition, the SphygmoCor system has been approved for clinical use by regulatory bodies such as the US FDA [12].

Systemic hemodynamic variables were measured simultaneously with the continuous pressure signals using the LiDCO™*plus* software (version 4.0, LiDCO Ltd., Cambridge, UK), which provides for a minimally invasive technique of hemodynamic monitoring. The methodology incorporates two methods, namely, a continuous arterial BP waveform analysis system (PulseCO), coupled to a single-point lithium indicator dilution calibration system (LiDCO) [26, 29]. The calibration procedure incorporates an injection of 0.3 mmol lithium chloride through a peripheral line [29]. The lithium is detected by an external lithium ion-sensitive external electrode connected to the peripheral arterial line, which enables monitoring of the hemodynamic variables cardiac output (CO), systemic vascular resistance (SVR), stroke volume (SV), cardiac index (CI), mean radial arterial BP, and heart rate (HR). The software in LiDCO™plus and Sensometrics® has an identical time reference. We used a minimally invasive approach to monitor the systemic hemodynamic variables, namely, the PulseCO hemodynamic monitor. This approach has previously been validated against the pulmonary artery catheter method [19] and applied for hemodynamic monitoring in several patient cohorts [2, 20].

**Fig. 2:**
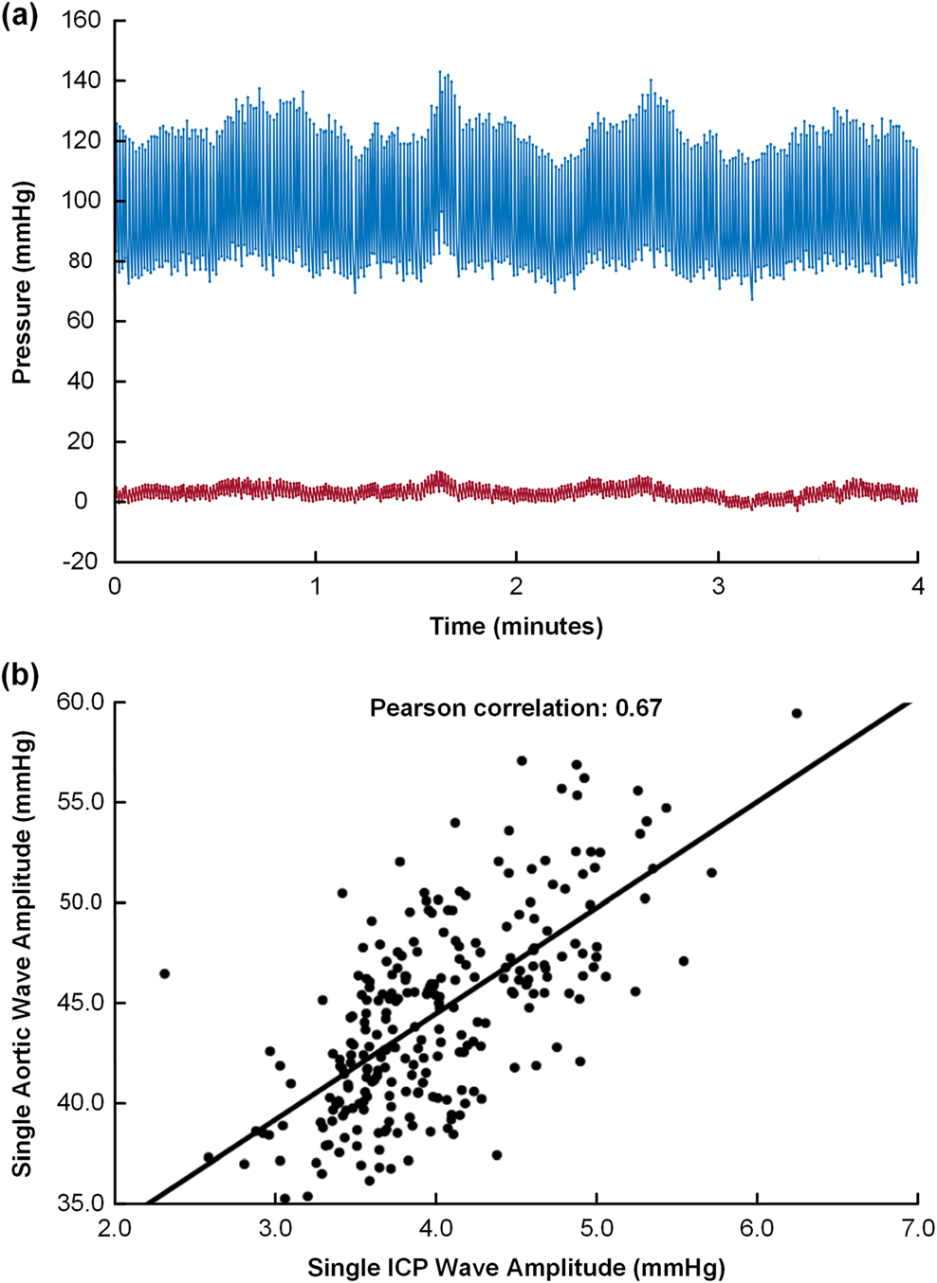
The intracranial aortic amplitude correlation. **a** Simultaneous central aortic blood pressure (BP; blue waveform) and intracranial pressure (ICP) single-pressure waves (red waveform) were automatically identified and the single aortic BP and ICP wave amplitude values determined for every aortic BP/ICP single wave pair. **b** The Pearson correlation coefficient of corresponding aortic BP/ICP single-wave amplitudes (intracranial aortic amplitude correlation, IAAC_AORTIC_) was determined for consecutive 4-min period. In this example, the Pearson correlation coefficient was 0.67 (*P* < 0.001). Notably, this example refers to one particular 4-min period; the magnitude of this IAAC_AORTIC_ observation was substantially higher than observed in the vast majority of 4-min periods

In this study, for the first time, the moving correlation between single-pressure wave amplitudes of ICP and central aortic BP waveform amplitudes were determined (Intracranial Aortic Amplitude Correlation; IAAC_AORTIC_). For this purpose, the peak to peak amplitudes of corresponding central aortic BP and ICP single pressure waves were automatically identified. In the following, the peak to peak amplitude refers to the difference between the maximum systolic BP and the minimum diastolic BP for the central aortic BP single waves and the difference between the maximum ICP and minimum ICP for the ICP single waves. The amplitude identification did not identify the P1, P2, and P3 peaks, but identified the highest peak between two diastolic minimum pressures. The intracranial aortic amplitude correlation (IAAC_AORTIC_) was thereafter determined as Pearson correlation coefficient for every consecutive 4-min period of the individual central aortic BP and ICP recording (Fig. 2). In previous studies, we have used a similar approach based on arterial BP measurements from the radial artery (intracranial arterial amplitude correlation; IAAC) [4, 10].

In the present study, we further explored the degree of intracranial aortic amplitude correlation for various levels of mean ICP wave amplitude (MWA_ICP_), systemic hemodynamic variables, and how the correlation related to various patient characteristics and to clinical response to CSF diversion surgery.

### Time alignment of pressure signals

As a central part of the study is to examine the correlation between the amplitudes of single waves resulting from the same heartbeat, perfect time alignment between the signals was crucial. To exclude uncertainties, time alignment of the time series was thoroughly checked, initially by visual comparison. If apparent artifacts due to patient movement did not align, or the time delay between signals shifted throughout the recording, there was reason to believe that there had been some corruption of the signal. In the dataset presented here, there was a systematically recurring time shift that only happened in one of the time series (central aortic BP waveforms). The time shift was corrected using a two-step approach. First, the onset of each single wave was calculated by a beat detection algorithm that utilizes a curve length transformation [38]. The onset before the anomaly was chosen for both time series and joined with the time aligned onset 6 s after, thereby removing the anomaly. The diastolic pressure over such a short timeframe was found to be approximately constant.

### Statistics

The statistical analyses were performed using the SPSS software version 25 (IBM Corporation, Armonk, NY). Differences between continuous data were determined using one-way ANOVA and Bonferroni post hoc tests for multiple comparisons. Statistical significance was accepted at the 0.05 level.

## Results

### Patients

The study included 29 patients with probable iNPH; demographic and management data are presented in Table 1. Twenty-two patients were shunted and seven managed conservatively. All iNPH patients had normal static ICP (mean ICP) < 18 mmHg, which is required for the diagnosis probable iNPH [33]. The iNPH patients responding clinically to CSF diversion surgery had elevated mean ICP wave amplitudes (*n* = 20; MWA_ICP_ = 5.8 mmHg), as compared with the nine iNPH patients managed conservatively or without clinical response to surgery (MWA_ICP_ = 3.5 mmHg; *P* < 0.001; independent samples *t* test).

**Table 1:** Material of patients with probable iNPH

iNPH patients (*N*)	29
Age (years)	75 ± 6 years
Gender (F/M)	15/14
BMI (kg/m^2^)	24.1 ± 3.7
Co-morbidity with arterial hypertension and/or diabetes mellitus (*N*)	11
Symptom duration (years)	2.5 ± 2.2
Severity of iNPH symptoms (iNPH grading scale^a^; median, ranges)	10 (7, 13)
Treatment	
CSF diversion surgery/no surgery (*N*)	22/7
Response to CSF diversion surgery	
Positive clinical response (Change in an iNPH grading scale^a^ > 1; *N*)	20
No positive clinical response (*N*)	2

^a^iNPH grading scale, ref. Eide and Sorteberg, 2010. Continuous data presented as average ± standard deviation

### The intracranial aortic amplitude correlation for different levels of mean ICP wave amplitude

The individual average levels of mean ICP wave amplitude (MWA_ICP_), determined from observations every 6 s, are presented in Table 2. With reference to our established thresholds [7], MWA_ICP_ was above threshold (> 4.0 mmHg) in 19/29 (66%) individuals. At the group level, the average MWA_ICP_ was 5.0 ± 1.8 mmHg (Table 2).

**Table 2:** Individual mean ICP wave amplitude (MWA_ICP_) vs. intracranial aortic amplitude correlation (IAAC_AORTIC_)

Patient	Mean ICP wave amplitude (MWA_ICP_)				Intracranial aortic amplitude correlation (IAAC_AORTIC_)			
	6-s observations (*N*)	Average (mmHg)	> 4.0 mmHg	< 4.0 mmHg	4-min observations (N)	Average	> 0.4	< 0.4
1	6764	3.2		1	103	0.33		1
2	7819	6.9	1		350	0.12		1
3	8114	3.3		1	325	0.47	1	
4	7532	5.1	1		325	0.32		1
5	4810	5.6	1		189	0.22		1
6	5280	5.5	1		238	0.40	1	
7	7226	5.0	1		119	0.29		1
8	4610	3.9		1	237	0.31		1
9	4757	3.6		1	237	0.25		1
10	6721	9.4	1		346	0.66	1	
11	6343	4.1	1		326	0.21		1
12	5762	3.0		1	233	0.03		1
13	7508	7.4	1		325	0.55	1	
14	5191	4.9	1		236	0.18		1
15	3933	3.9		1	194	0.03		1
16	4892	4.8	1		301	0.12		1
17	5704	3.6		1	264	0.37		1
18	4021	4.4	1		208	0.10		1
19	8682	4.1	1		234	0.12		1
20	5681	10.9	1		267	0.16		1
21	4918	5.6	1		222	0.59	1	
22	4172	3.9		1	260	0.34		1
23	4136	6.2	1		223	0.15		1
24	8161	5.3	1		357	0.30		1
25	8086	4.6	1		327	0.44	1	
26	6350	3.3		1	207	0.22		1
27	6492	6.2	1		244	0.21		1
28	5506	2.9		1	255	0.17		1
29	4487	5.5	1		208	0.39		1
AVG ± STDEV	5988 ± 1439	5.0 ± 1.8			254 ± 64	0.28 ± 0.16		
*N*			19	10			6	23

The individual average values for intracranial aortic amplitude correlation (IAAC_AORTIC_) over consecutive 4-min periods are shown in Table 2. The average number of 4-min periods available for analysis for individuals of this cohort was 254 ± 64. At the group level, the average intracranial aortic amplitude correlation (IAAC_AORTIC_) was 0.28 ± 0.16 (Table 2). While a threshold level for IAAC_AORTIC_ has previously not been established, given a threshold of IAAC_AORTIC_ > 0.4, 6/29 (21%) of individuals were above threshold.

**Fig. 3:**
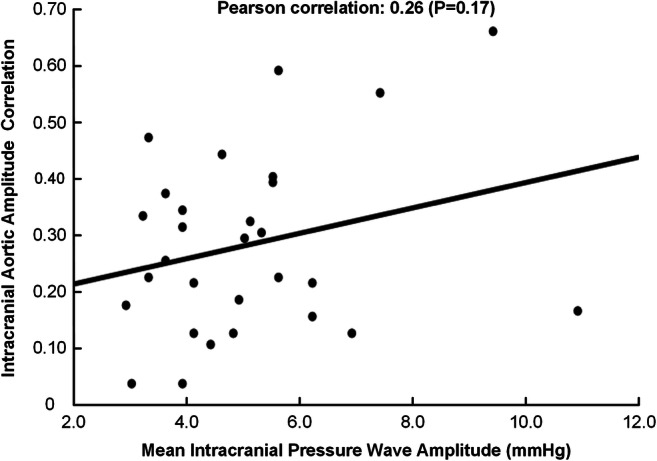
Association between intracranial aortic amplitude correlation and the level of mean ICP wave amplitude. The IAAC_AORTIC_ is plotted for different levels of MWA_ICP_, including presentation of the fit line and the Pearson correlation coefficient (R) with *P* value

Among the 19 individuals with MWA_ICP_ > 4.0 mmHg, IAAC_AORTIC_< 0.4 was observed in 14 (74%) (Table 3). Moreover, among the 10 patients with MWA_ICP_ < 4.0 mmHg, one individual (10%) had IAAC_AORTIC_ > 0.4. Even though MWA_ICP_ tended to be higher when IAAC_AORTIC_ increased, this was not significant (*P* = 0.30; Pearson chi-square test). Defining a threshold of IAAC_AORTIC_ of 0.3 gave no different results (*P* = 0.68; Pearson chi-square test). We also determined the association between individual average levels of IAAC_AORTIC_ and MWA_ICP_ that was non-significant (Fig. 3).

**Table 3:** Number of individuals with MWA_ICP_/IAAC_AORTIC_ combinations above or below thresholds

		MWA_ICP_ (mean ICP wave amplitude)	
		> 4.0 mmHg	< 4.0 mmHg
IAAC_AORTIC_ (intracranial aortic amplitude correlation)	> 0.4	5	1
	< 0.4	14	9

*P* = 0.30 (Pearson chi-square test)

### The intracranial aortic amplitude correlation versus systemic hemodynamic variables

**Fig. 4:**
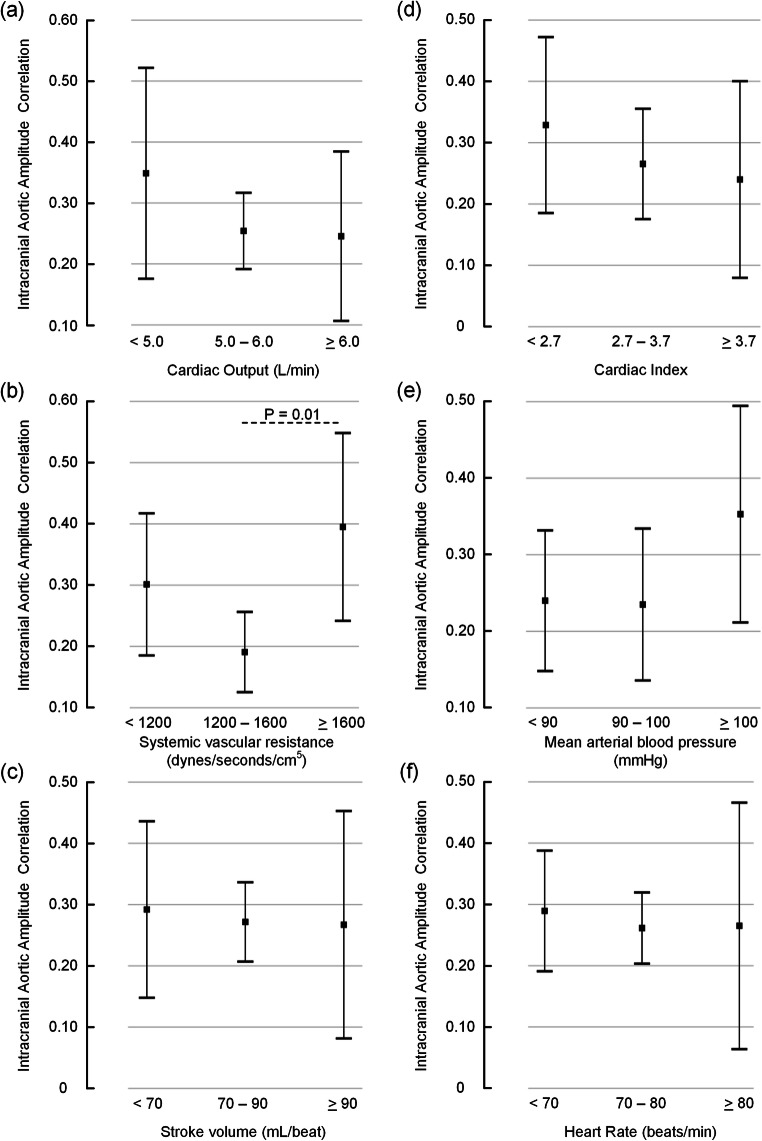
The intracranial aortic amplitude correlation for different categories of systemic hemodynamic parameters. The level of intracranial aortic amplitude correlation (IAAC_AORTIC_) is presented for different categories of **a** cardiac output, **b** systemic vascular resistance, **c** stroke volume, **d** cardiac index, **e** mean arterial blood pressure, and **f** heart rate. Each error bar is presented as mean with 95% CI. Except for significantly different IAAC_AORTIC_ between the categories with systemic vascular resistance 1200–1600 or > 1600 dynes/s/cm^5^ (**b**), there were no significant differences between groups (ANOVA with post hoc Bonferroni tests)

The intracranial aortic amplitude correlations (IAAC_AORTIC_) present in Table 2 were assessed for various levels of the systemically measured hemodynamic variables. Since the IAAC_AORTIC_ is an index derived from a correlation coefficient, we decided to assess categories of the systemic hemodynamic variables. The correlation IAAC_AORTIC_ was significantly higher in the group with systemic vascular resistance (SVR) above 1600, compared to the group with SVR in the range 1200–1600 dynes/s/cm^5^. However, the degree of correlation was not different for other levels of the systemic hemodynamic variables. Figure 4 presents average values of IAAC_AORTIC_ for various levels of CO (Fig. 4a), SVR (Fig. 4b), SV (Fig. 4c), CI (Fig. 4d), mean arterial BP (Fig. 4e), and HR (Fig. 4f).

### The intracranial aortic amplitude correlation versus patient characteristics

**Fig. 5:**
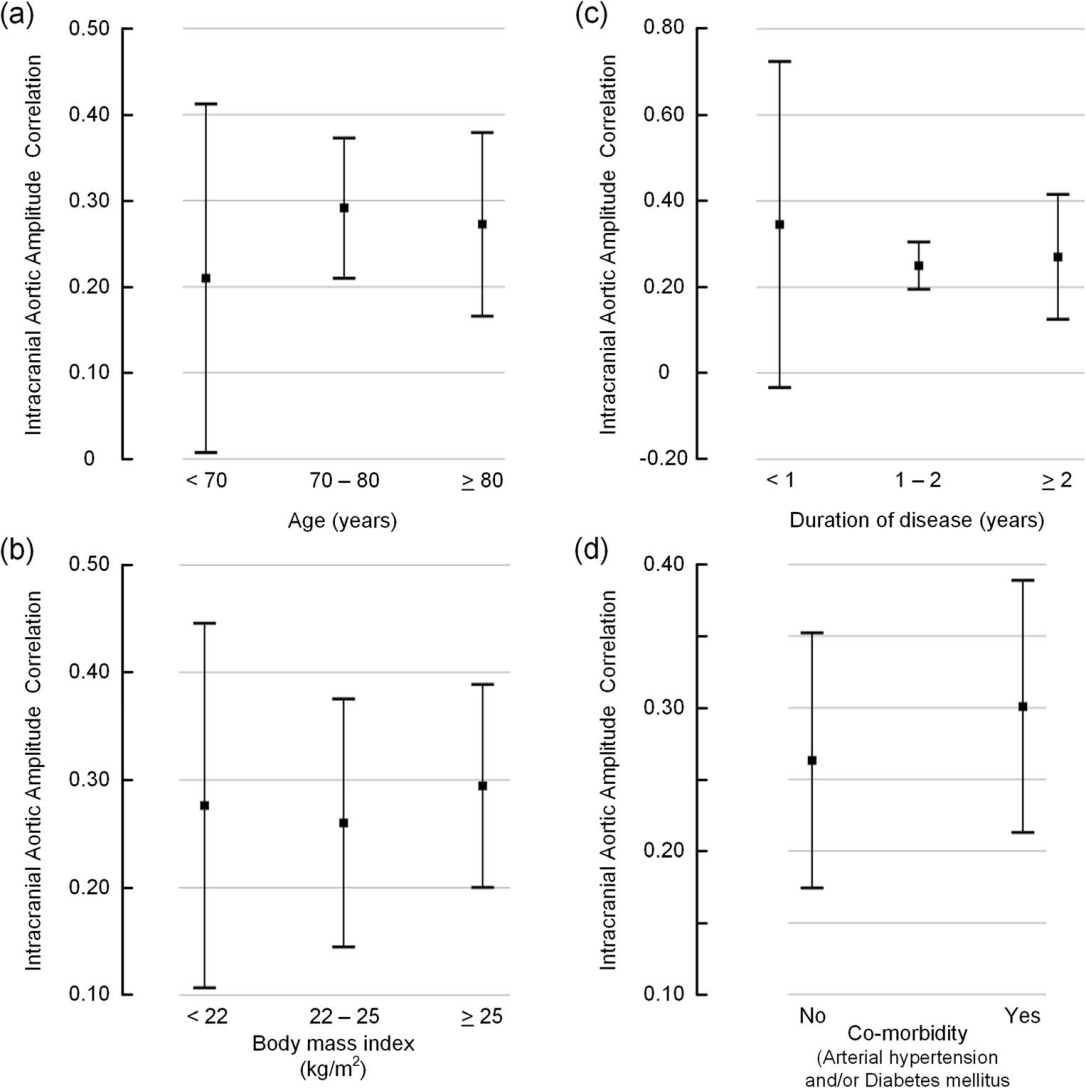
The intracranial aortic amplitude correlation for different categories of patient characteristics. The level of intracranial aortic amplitude correlation (IAAC_AORTIC_) is presented for different categories of patient data, including **a** age, **b** body mass index (BMI), **c** duration of disease, and **d** presence of co-morbidity (i.e., arterial hypertension and/or diabetes mellitus). Each error bar is presented as mean with 95% CI. There were no significant differences between groups (ANOVA with post hoc Bonferroni tests)

**Fig. 6:**
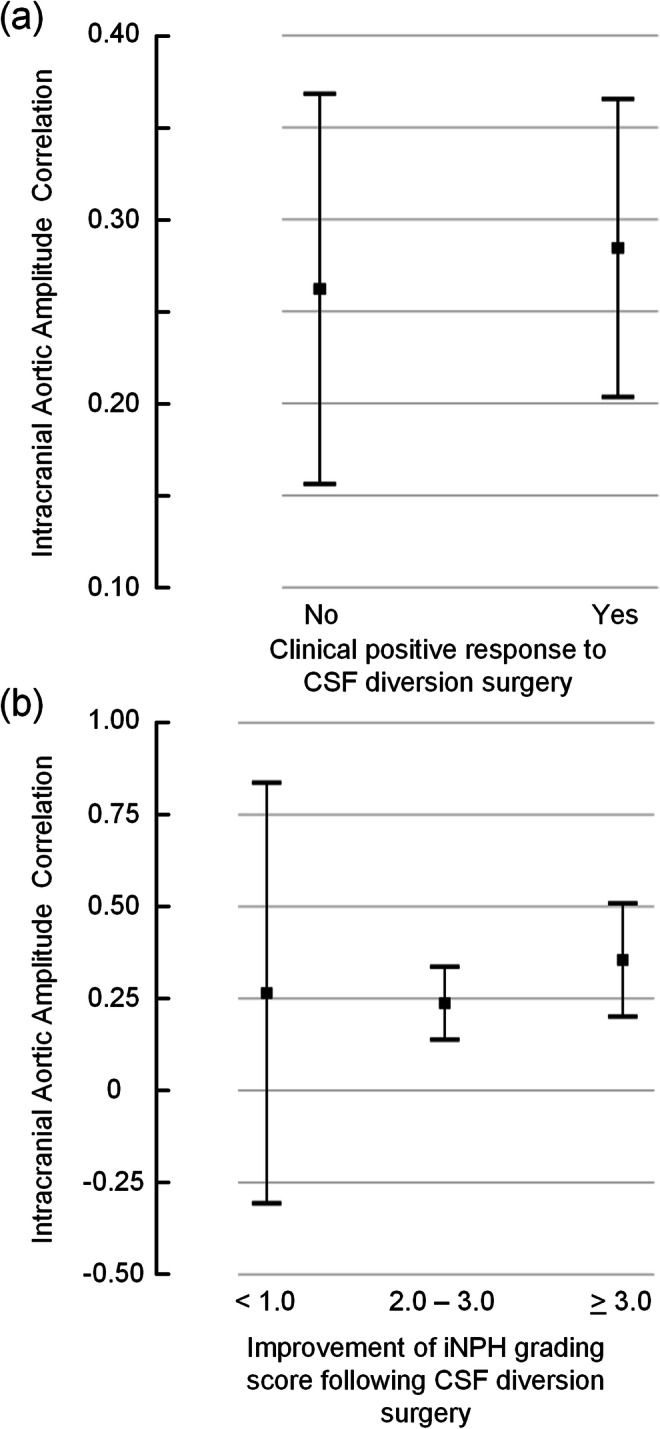
The intracranial aortic amplitude correlation for different categories of clinical response to CSF diversion surgery. The level of intracranial aortic amplitude correlation (IAAC_AORTIC_) is presented for **a** the sub-groups of individuals with either no positive clinical response or a positive clinical response to CSF diversion surgery, and **b** for sub-groups with different degree of clinical response to CSF diversion surgery. Each error bar is presented as mean with 95% CI. There were no significant differences between groups (ANOVA with post hoc Bonferroni tests, and revealed)

It could be expected that patient characteristics substantially modify the intracranial aortic amplitude correlation. However, as shown in Fig. 5, we found no difference in IAAC_AORTIC_ for various categories of age (Fig. 5a), BMI (Fig. 5b), duration of disease (Fig. 5c), or presence of co-morbidity (i.e., arterial hypertension and/or diabetes mellitus; Fig. 5d). Another point worth investigating is whether IAAC_AORTIC_ is dependent on the clinical response to CSF diversion (shunt) surgery. We found no evidence that neither shunt response (Fig. 6a) nor the degree of shunt response (Fig. 6b) was related to the level of IAAC_AORTIC_ in this study.

## Discussion

The main observation was a rather low degree of correlation between intracranial and aortic pressure amplitudes at the group level (IAAC_AORTIC_ average of 0.28 ± 0.16), though a higher degree of correlation (> 0.4) was seen in about 1/5 iNPH patients. Moreover, the correlation was hardly affected by systemic hemodynamic variables, except for in a subgroup with increased systemic vascular resistance.

The presently reported elevated mean ICP wave amplitudes in iNPH patients responding favorably to CSF diversion surgery confirm previous clinical experience from larger iNPH patient cohorts [7, 8].

In the past, the relationship between radial arterial BP and ICP pressure signals has been studied extensively in the frequency domain and been referred to as transfer function or systems analysis [27, 28]. These studies were interpreted to provide evidence that loss of vasomotor tone of the precapillary vessels changed the radial arterial BP to ICP transmission into a passive and linear pressure transmission [28, 30, 31]. As loss of vasomotor tone is an indication of impaired autoregulation, this implies that the physical mechanisms that dampen parts of the frequency spectrum are reduced or diminished when autoregulation is reduced. As a result, the correlation between mean radial arterial BP and mean ICP was established as a surrogate marker of intracranial pressure autoregulation [36]. When autoregulation is impaired, the physical mechanisms dampen less, and the correlation increases. While comparing mean levels, the correlation is denoted the cerebrovascular Pressure-Reactivity index (PRx). A similar index exists for the correlation between single-pressure wave amplitudes of ICP and radial arterial BP [1, 11].

The current study explored a comparable concept to that explained in the previous work [27, 28], but differs from previous studies by utilizing the central aortic BP waveform for the first time. A limitation with the studies utilizing radial artery BP measurements for IAAC estimation [4, 10, 11] is that the radial artery is more peripheral to the brain than the aortic artery. A criticism against use of radial artery measurements is that the BP measurements are too far from the intracranial compartment. In this regard, we would expect a closer association between central aortic BP waveforms and ICP waveforms making central aortic BP estimates more relevant.

While previous studies have primarily addressed the role of cerebrovascular factors on the ICP waveform, we here aimed at focusing on both the cerebrovascular and the extra-cerebrovascular factors. The latter causes physical filtering of the intracranial arterial BP waveform (see Fig. 1). Using the wave amplitude as the primary waveform characteristic, we investigated the role played by the source (the arterial BP waveform) and the filter (the intracranial constituents). Intracranial aortic amplitude correlation values approaching 0 indicate low degree of association, indicating that the ICP waveform is less impacted by vascular factors (preserved autoregulation) and that is primarily determined by extra-cerebrovascular factors (i.e., alterations in the brain and CSF). Correlation values approaching + 1, on the other hand, implies a direct association between alterations in arterial BP and ICP, which suggest a more extensive role of vascular factors such as impaired pressure autoregulation and altered cerebral blood flow.

There are presently no established threshold values for which intracranial aortic amplitude correlation values represent an upper threshold value. We would expect this correlation to be higher than previously reported correlation levels that were based on peripheral arterial BP measurements. In comparison, despite the general agreement that the traditional PRx and IAAC indices of cerebrovascular pressure-reactivity can be looked upon as surrogate markers of intracranial pressure autoregulation, the threshold levels for impairment/not impairment have not been defined. A clinical study showed that the outcome seems to worsen when PRx remained above 0.2–0.3 [39] among a cohort of individuals with traumatic brain injury. A different study reported that average values of amplitude correlation above 0.2 during week 1 after a subarachnoid hemorrhage was associated with worse outcome [11]. The thresholds for impaired autocorrelation are thereby clearly in the lower part of the spectrum.

In the present study, the average correlation between ICP and central aortic BP amplitudes was low in our cohort (IAAC_AORTIC_ 0.28 ± 0.16) and hardly influenced by the systemic hemodynamic variables. However, in 6/29 of the patients (21%), the average correlation IAAC_AORTIC_ was above 0.40. This might indicate that cerebrovascular factors play a dominating role in determining the ICP wave amplitude level in this subgroup. The cerebrovascular factors may be impaired cerebral pressure autoregulation as well as cerebrovascular disease that together affect cerebral blood flow and thereby the ICP wave amplitudes. Cardiovascular risk factors are more prevalent in iNPH; the prevalence of arterial hypertension and diabetes is increased in patients with iNPH [5]. This fits well with our findings of increased correlation IAAC_AORTIC_ for a subgroup with high systemic vascular resistance. Another aspect is that BP waveforms change with age. Notably, iNPH is a disease of the elderly. Elevated ICP wave amplitudes in these individuals could be an age phenomenon. In line with this assumption, a study by Lloyd et al. [22] reported an age-dependent change in the intracranial arterial waveform that corresponded well with the arterial wall stiffening seen with increased age. However, we found no increase in IAAC_AORTIC_ with increasing age in our cohort. If the intracranial arterial BP waveform was a decisive factor for pulsatile ICP, we would, therefore, expect an age-dependent IAAC_AORTIC_, as the amplitude is the major waveform characteristic.

Among the present 19 patients with ICP wave amplitudes above the threshold (MWA_ICP_ > 4.0 mmHg), 5/19 individuals (26%) also presented with IAAC_AORTIC_ indices above 0.4. According to our model, the levels of ICP wave amplitudes in this subgroup might be partly affected by cerebrovascular factors such as impaired autoregulation. However, in the majority of the patients with iNPH, the levels of ICP wave amplitudes seemed to be primarily determined by extra-cerebrovascular factors. Accordingly, in 74% of iNPH individuals, the increased ICP wave amplitudes were accompanied with IAAC_AORTIC_ below 0.4. Moreover, at the group level, the intracranial aortic amplitude correlation was not different for various levels of mean ICP wave amplitude (Fig. 3). On this background, we find it difficult to explain elevated ICP wave amplitudes by altered intracranial arterial BP amplitudes and suggest it is necessary to look at other possible causes of elevated pulsatile ICP in the majority of iNPH patients. In this regard, the recently described glymphatic system for transport of fluid and solutes in the central nervous system [15] could be particularly relevant. The glymphatic system may play a critical role in the brain’s ability to remove toxic metabolic waste products [32], and glymphatic magnetic resonance imaging (gMRI) gave evidence of impaired glymphatic function in iNPH patients [6, 34]. In rodents, reduced arterial pulsations, such as seen in arterial hypertension, were associated with hampered antegrade transport of fluid and solutes along the blood vessels [24]. Likewise, we hypothesize that unfavorable properties of the extra-cerebrovascular compartment may cause restriction of intracranial arterial BP pulsations and result in impaired glymphatic circulation [6]. In histopathological studies of brain tissue specimens of iNPH subjects, astrogliosis has been found, which may induce stiffening of the brain, as well as a loss of perivascular water channels aquaporin-4, which may hamper glymphatic circulation [13]. Accordingly, processes at the glia-vascular interface may be extra-cerebrovascular factors responsible for alterations in pulsatile ICP. Future studies addressing disease processes affecting the extra-cerebrovascular compartment in iNPH may lay the basis for medical treatment of this dementia disease.

## Limitations

The present approach aiming at differentiation between cerebrovascular and extra-cerebrovascular factors represents a simplification, as these factors interact in vivo. Moreover, the cerebrovascular factors incorporate several variables such as cerebral blood flow changes, vascular wall alterations, and cerebrovascular tone-related variations in autoregulation. Likewise, the extra-cerebrovascular factors may involve various alterations in the brain parenchyma and its interaction with CSF. Nevertheless, the study of complex physiological mechanisms requires simplification. In this regard, the differentiation between cerebrovascular and extra-cerebrovascular factors seems one useful approach.

The previous studies examining the moving correlation between ICP and arterial BP waveform amplitudes exclusively utilized peripheral arterial BP measurements, typically from the radial artery [4, 9, 10]. We hypothesized that central aortic BP estimates are better for this purpose. It should be noted, however, that despite the thorough validation, the central aortic BP waveforms used here are indeed estimates. They thereby do provide an additional source of uncertainty in the analysis and are not the perfect proxy for intracranial arterial BP waveforms. The SphygmoCor systems extensive validation study, however, does provide some reassurance of the validity of the estimates [12]. A preliminary study showing a higher similarity between ICP waveforms and central aortic BP waveforms compared to radial arterial BP waveforms further substantiates our observations [18]. Various other epidemiological [21, 35] and clinical studies [23, 37] using the SphygmoCor system supports the same conclusion.

## Conclusions

In about 1/5 iNPH patients of this study, the intracranial aortic amplitude correlation (IAAC_AORTIC_) was rather high (average Pearson correlation coefficient > 0.4), suggesting that cerebrovascular factors to some extent may affect the ICP wave amplitudes in a subset of patients. However, in 14/19 (74%) iNPH patients with elevated ICP wave amplitudes, the intracranial aortic amplitude correlation was low, indicating that the ICP pulse amplitude in most iNPH patients is independent of central vascular excitation, ergo it is modulated by local cerebrospinal physiology. In support of this assumption, the intracranial aortic amplitude correlation was not related to most systemic hemodynamic variables. An exception was found for a subgroup of the patients with high systemic vascular resistance, where there was a correlation.
